# Society Meetings

**Published:** 1914-01

**Authors:** 


### SOCIETY MEETINGS.

*Brief reports and announcements of meetings of societies of Western New York and of those of general scope are requested from Secretaries. Copy should be on hand by the fifteenth of each month. Full transactions will be published at cost of composition.*

The Dunkirk and Fredonia Medical Society held its annual meeting, preceded by a banquet, at the Hotel Gratiot, Dunkirk, Dec. 10. Dr. G. E. Smith of Fredonia presented a paper. The following officers were elected: President, Dr. Walter H. Vosburg; Vice President, Dr. N. E. Beardsley; Secretary and Treasurer, Dr. William J. Sullivan, all of Dunkirk.

The Wellsville Medical Club has been organized for the purpose of studying local sanitary problems. Monthly meetings will be held. The officers are Dr. F. E. Comstock, President, and Dr R. M. Eaton, Secretary-Treasurer.

---

The Medical Society of the County of Chemung held its annual meeting in its rooms in the Federation Building, Elmira, Dec. 16. The program was as follows:

Prolapse of the Uterus.....................N. H. Soble, M. D.
Medical Economics.........................R. G. Loop, M. D.
Glaucoma.....................................C. L. Carey, M. D.
Some Personal Observations on Smallpox....F. B. Parke, M. D.

---

Buffalo Academy of Medicine. Nov. 25, 1913, Section of Pathology; Syphilis of the Meninges, Dr. E. R. Le Count, Prof. of Pathology, Rush Medical College, Chicago.

Dec. 2, Section of Surgery; Drs. Thew Wright and Charles W. Bethune of Buffalo, Demonstration of Foreign Bodies in the Bladder, Dr. Lincoln Davis of Boston, Calculus Anuria.

Dec. 9, Section of Medicine; Dr. Richard C. Cabot of Boston, Diagnosis of Diseases Causing Epigastric Pain. This was in the nature of an informal talk, with blackboard tabulation, the speaker raising many questions of interest for future solution. Dr. Cabot also gave an afternoon clinic at the General Hospital. Dr. John H. Pryor of Buffalo presented specimens from a case of high cancerous obstruction of the oesophagus, with abscess forming secondarily. The case report will be published later in this Journal.

Dec. 16, Section of Obstetrics and Gynaecology; Dr. F. C. Goldsborough presented a specimen from a case of Perforation of Pregnant Uterus with Curette; Dr. Thomas H. McKee spoke on "New Wine in Old Bottles"; Dr. James E. King gave a lantern slide demonstration of The Present Status of Operative Treatment of Cervical Cancer. The discussion was opened by Dr. Herman E. Hayd (all of Buffalo).

---

The Medical Society of the County of Chautauqua held its annual meeting at Dunkirk, Dec. 9, 1913. Dr. N. G. Richmond of Fredonia delivered the President's address, "The Functions of a County Medical Society"; Dr. A. L. Benedict of Buffalo read a paper on Hyperchlorhydria—A Paradoxic Disease; Dr. H. S. Reger of Jamestown on Mouth Breathing and Its Correction and Dr. William J. Sullivan of Dunkirk on the Pituitary Bodies. The last will be published in this Journal. Dr. George F. Smith of Falconer was elected President.

Medical Society of the County of Monroe. Election of officers Dec. 16, 1913: President, Albert C. Snell; Vice-President, F. W. Zimmer; Secretary, C. W. Hennington; Treasurer, F. W. Seymour; Board of Censors, C. E. Darrow, E. H. Howard, R. M. Moore, C. R. Witherspoon, J. F. W. Whitbeck. Delegates to State Society, two for term of two years, L. W. Howk and W. E. Bowen; alternates, M. B. Palmer and J. M. Swan. Delegates to Seventh Dist. Branch, one for two years, W. W. Percy; alternate, W. H. Sutherland. Members of Milk Commission, two for three years, J. W. Magill and E. G. Nugent.

---

The National Conference on Race Betterment will meet at Battle Creek, Jan. 8-12, 1914. A long and interesting program, including the presentation of moving pictures, models and demonstrations, has been provided. There are no dues, but $1 will be charged for the printed proceedings. The Battle Creek Sanitarium will act as host for the Conference. The President is Dr. Stephen Smith, Vice President of the N. Y. State Board of Charities.

---

The Niagara Frontier Organization has been formed to urge the improvement of the Niagara River Road as a boulevard. We expressed our opinion of the present boulevard in the November issue. Mayor John A. Rafter of North Tonawanda, Hon. Peter A. Porter and George F. Nye of Niagara Falls addressed the initial meeting at La Salle, Dec. 22.

---

The Rochester Academy of Medicine, Section on Public Health, met December 10 in the Academy Rooms, 355 East Ave. Dr. C. C. Sutter gave a paper on the Development of Complement Fixation Tests; Dr. C. O. Boswell on The Wassermann Reaction—Some Conclusions; Dr. Raymond Sanderson of Canandaigua on the same in Diagnosis and Prognosis. The discussion was opened by Drs. Joseph Roby and E. Wood Ruggles.

---

The ninety-second annual meeting of the Medical Society of the County of Erie was held in the Buffalo Library Building on Monday evening, December 15, 1913, President Whitwell presiding. The minutes of the previous regular meeting and of the council meetings were read and approved.

The following new members were duly elected: Dr. Edward C. Koenig, Dr. Hugh C. McDowell, Dr. Gaetano Rescigno, Dr. Geo. P. Michel, Dr. Lucy A. Kenner, and Dr. Wm. L. Howard was elected by transfer from the Medical Society of the County of Monroe.

President Whitwell appointed Drs. Kauffmann and Phillips as tellers and Treasurer Lytle as clerk for the annual election.

President Whitwell called Vice-President Woodruff to the chair and then delivered his annual address.

Annual reports were submitted by the Board of Censors through Dr. J. D. Bonnar; by the Committee on Public Health, through Dr. H. R. Hopkins; by the Committee on Psychopathic Hospital and Examination in Lunacy, through Dr. F. S. Crego; by the Committee on Collection of Accounts, through Dr. Wm. H. Thornton; by the Milk Commission, through Dr. Clayton W. Greene; by the Committee on Contract Practice, through Dr. Edward E. Haley, and the Annual Report of the Treasurer, by Dr. A. T. Lytle.

The tellers then presented their report of the election, and the following officers were declared duly elected:

President—Dr. John V. Woodruff.

First Vice President—Dr. Arthur W. Hurd.

Second Vice President—Dr. Franklin W. Barrows.

Secretary—Dr. Franklin C. Gram.

Treasurer—Dr. Albert T. Lytle.

Censors—Drs. John D. Bonnar, Francis E. Fronczak, Arthur D. Bennett, Irving W. Potter and Archibald D. Carpenter.

Chairman Committee on Legislation—Dr. F. Park Lewis.

Chairman Committee on Public Health—Dr. Henry R. Hopkins.

Chairman Committee on Membership—Dr. Grover W. Wende.

Delegates to the State Society—Drs. Albert T. Lytle, Julius Ullman, Julius Richter and Franklin W. Barrows.

Dr. Whitwell then turned over the chair to President-elect Dr. Woodruff, who briefly thanked the Society for the honor conferred upon him, after which a rising vote of thanks was extended to retiring President Whitwell for the able manner in which he had presided during the past year.

The Council was empowered to make arrangements for the visit in January of President Witherspoon of the A. M. A.

